# Plasma hPG_80_ (Circulating Progastrin) as a Novel Prognostic Biomarker for early-stage breast cancer in a breast cancer cohort

**DOI:** 10.1186/s12885-023-10729-1

**Published:** 2023-04-04

**Authors:** Alexandre Prieur, Andrew Harper, Momtafin Khan, Bérengère Vire, Dominique Joubert, Léa Payen, Karen Kopciuk

**Affiliations:** 1 Biodena Care, 2040 Avenue du Père Soulas, 34090 Montpellier, France; 2 Cancer Epidemiology and Prevention Research, Alberta Health Services, 2210 – 2 Street SW, Calgary, AB T2S 3C3 Canada; 3 Lyon Sud Hospital, 69310 Pierre-Benite, France; 4 Departments of Oncology, Mathematics and Statistics, Community Health Sciences, University of Calgary, 2500 University Drive NW, Calgary, AB T2N 1N4 Canada

**Keywords:** Breast cancer, Circulating progastrin, hPG_80_, Prognostic biomarker, Recurrence, Survival

## Abstract

**Background:**

Recurrence and metastases are still frequent outcomes after initial tumour control in women diagnosed with breast cancer. Although therapies are selected based on tumour characteristics measured at baseline, prognostic biomarkers can identify those at risk of poor outcomes. Circulating progastrin or hPG_80_ was found to be associated with survival outcomes in renal and hepatocellular carcinomas and was a plausible prognostic biomarker for breast cancer.

**Methods:**

Women with incident breast cancers from Calgary, Alberta, Canada enrolled in the Breast to Bone (B2B) study between 2010 to 2016 and provided blood samples prior to any treatment initiation. Plasma from these baseline samples were analysed for circulating progastrin or hPG_80_. Participant characteristics as well as tumour ones were evaluated for their association with hPG_80_ and survival outcomes (time to recurrence, recurrence – free survival, breast cancer specific survival and overall survival) in Cox proportional hazards regression models.

**Results:**

The 464 participants with measurable hPG_80_ in this study had an average age of 57.03 years (standard deviation of 11.17 years) and were predominantly diagnosed with Stage I (52.2%) and Stage II (40.1%) disease. A total of 50 recurrences and 50 deaths were recorded as of June 2022. In Cox PH regression models adjusted for chemotherapy, radiation therapy, cancer stage and age at diagnosis, log hPG_80_ (pmol/L) significantly increased the risks for recurrence (Hazard Ratio (HR) = 1.330, 95% Confidence Interval (CI) = (0.995 – 1.777, *p* = 0.054)), recurrence-free survival (HR = 1.399, 95% CI = (1.106 – 1.770), *p* = 0.005) and overall survival (HR = 1.385, 95% CI = (1.046 – 1.834), = 0.023) but not for breast cancer specific survival (HR = 1.015, 95% CI = (0.684 – 1.505), *p* = 0.942).

**Conclusions:**

hPG_80_ levels measured at diagnosis were significantly associated with the risk of recurrence or death from any cause in women with breast cancer. Since the recurrence rates of breast cancer are still relatively high amongst women diagnosed at an early stage, identifying women at high risk of recurrence at their time of diagnosis is important. hPG_80_ is a promising new prognostic biomarker that could improve the identification of women at higher risk of poor outcomes.

## Background

Breast cancer is not only the most common cancer amongst Canadian women, but is also the second leading cause of cancer-related deaths in Canadian women as well [1]. The 5-year net survival rates for women with breast cancer is 89% depicting a generally favourable prognosis of breast cancer due to early detection from breast cancer screening and modern treatments [1]. Currently, appropriate treatments for breast cancer are determined by the use of predictive markers [2, 3]. Predictive biomarkers allow clinicians to determine the best course of treatment depending on the type of breast cancer the woman has [3]. The most common breast cancer biomarkers are estrogen receptors (ER), progesterone receptors (PR), and Human Epidermal Growth Factor Receptor 2 (HER2) [2]. After determination of which receptors are positive, the breast cancer subtype is identified which affects the treatment options [2].

Prognostic biomarkers can supplement this information from predictive biomarkers and provide additional insights to clinicians on which course of treatment is most appropriate in the long term [3, 4]. Prognostic biomarkers indicate the aggressiveness, invasiveness, and extent of spread of tumors which can aid in determining the recurrence risks and even survival outcomes [3]. Traditional prognostic biomarkers include axillary lymph node status, tumor size and grade, age at diagnosis, and nuclear and histological grade [3, 4]. The most common prognostic blood-biomarkers used for breast cancer include cancer antigen 15–3 (CA 15–3), carcinoembryonic antigen (CEA), HER2, and mucin 1 [5]. A systemic review revealed that while these prognostic biomarkers are useful in the entire breast cancer population, their performance was suboptimal in young and elderly patient groups [4]. The combined use of predictive and prognostic biomarkers enables clinicians to select more individualized treatments for breast cancer patients. With increasing knowledge on the pathophysiology of breast cancer, identifying biomarkers that can provide more individualized and targeted therapy for women with breast cancer are still needed, especially those identifying aggressive disease.

Although earlier stages of breast cancer have a more favourable prognosis [6], approximately 20–30% of early stage breast cancer patients develop bone metastasis which is the most frequent site (about 70%) for breast cancer metastasis [7]. Local recurrence of breast cancer occurs in 8% to 10% of patients [8]. Unfortunately, bone metastasis is not curable with women experiencing bone-only metastasis having a median overall survival ranging from 3–5 years [9, 10]. The Canadian Cancer Society statistics suggest that of 78 Canadian women diagnosed with breast cancer, 15 will die from it [1]. Thus, identifying women at risk of disease recurrence, metastases or death at the time of diagnosis are needed to improve these outcomes.

A novel blood-based biomarker that has shown promise in several different types of cancer is circulating progastrin or hPG_80_. It has been detected at significantly higher concentrations in the blood of cancer patients than in healthy blood donors [11, 12]. In physiology, progastrin is the precursor of gastrin synthetized by antrum G cells and processed into gastrin [13]. As a consequence, progastrin is barely detectable in the blood of healthy subjects [14]. hPG_80_ was initially studied in colorectal cancer and was found to be released in the blood stream from tumor cells, promoting carcinogenic activities [11, 14, 15]. In tumor cells, the *GAST* gene, which encodes hPG_80_, is a direct target gene of the WNT/β-catenin oncogenic pathway which is activated in many cancers [16]. hPG_80_ is directly associated with tumor cells survival [11, 15, 17, 18]. More recently, in both renal and hepatocellular cancers, hPG_80_ has shown a significant association with survival for both cancers [19, 20]. The hepatocellular cancer patients showed a higher sensitivity to hPG_80_ than alpha-fetoprotein (AFP) – the standard available diagnostic marker for hepatocellular cancer [20]. Thus, the plausibility of utilizing hPG_80_ as a blood prognostic biomarker for breast cancer exists.

Previous research has shown the clinical utility of hPG_80_ to detect patients at risk for poor survival outcomes, even amongst those with early-stage disease [20]. So, its utility in a breast cancer cohort established to identify prognostic biomarkers was a unique opportunity to evaluate it in this patient group. This study evaluated the association of hPG_80_ with a variety of outcomes including disease recurrence, recurrence-free survival, breast cancer specific survival and overall survival among a cohort of breast cancer patients from the breast-to-bone (B2B) cohort.

## Methods

### Study population

The B2B Metastasis research program interviewed and recruited 478 women between 2010 to 2016 who met the eligibility criteria of having incident primary breast cancer (stage I-IIIc) at baseline, with no prior history of cancer (except for cervical in-situ neoplasia and non-melanoma skin cancer), who were between the ages 18 to 80 years and were residents of Calgary, Alberta, Canada and the surrounding areas. After diagnosis but before surgery or any treatments began, 471 participants provided blood samples of sufficient quantity and quality at a provincial laboratory location. Samples were transferred to the Alberta Cancer Research Biorepository (ACRB) for storage in -80 °C freezers. Patients were also followed-up at 24, 48, and 72-month intervals post-diagnosis and were asked to complete self-administered follow-up Health and Lifestyle questionnaires, Canadian Diet History Questionnaires I/II, and Past Year Physical Activity Questionnaire; blood samples were also collected at these time points. Further information on the study population and recruitment can be found in the baseline paper [21].

### hPG_80_, participant and clinical variables

A 500 μl aliquot of EDTA plasma from 471 participants was retrieved from the ACRB inventory and were couriered on dry ice to the biology and pathology center (Les Hospices Civils de Lyon, France) on November 22, 2021 where the hPG_80_ levels were then measured using the DxPG_80_ lab kit (Biodena care). The analytical performances of the kit are described in Cappellini et al. [22]. Briefly, the limit of detection (LoD) is 1 pmol/L and the limit of quantitation (LoQ) is 3.3 pmol/L. The inter- and intra-assay coefficients of variation (CV%) were below 10%. No cross-reactivity was detected with gastrin-17, Gastrin-Gly or CTFP (C-Terminus Flanking Peptide). No cross-reactivity was detected with other blood biomarkers such as CA125, CEA or PSA. No interference was detected with chemicals such as SN-38, 5-FU or triglycerides, cholesterol or hemoglobin [20]. hPG_80_ values for 464 participants were successfully obtained, although 129 of them had values below the assay’s LoQ threshold.

A Health Records Technician with Alberta Health Services, the provincial health authority in Alberta, carried out chart reviews for 120 B2B participants identified to be at the highest or lowest risk of breast cancer recurrence using an algorithm based on administrative data [23]. Vital status was updated through linkage with the Alberta Cancer Registry (ACR), where dates and causes of death (if known) were obtained up to December 2021. Participant factors considered in this study included age at diagnosis and menopausal status recorded at baseline. Menopausal status was imputed for 35 for the 464 women who comprised this study population. Women older than 50 years of age or those who had a history of hysterectomy or oophorectomy at any age were deemed to be post-menopausal (22 women); the remaining ones were deemed to be pre-menopausal (13 women). Tumour characteristics measured at baseline included stage, grade, size and hormonal statuses. Treatment factors included chemotherapy, radiotherapy, immunotherapy and hormonal therapy. All participants had surgery to remove their tumours.

### Statistical methods

Statistical methods included descriptive statistics of the clinical and demographic variables from the study population, including hPG_80_ levels. Survival events included breast cancer recurrence (local or distant), and death from any cause or from breast cancer. Survival outcomes were based on the time elapsed from diagnosis to the first event and included disease-free survival (first occurrence of death or recurrence), overall survival (death from any cause), breast cancer-specific survival (death from breast cancer), and time to breast cancer recurrence. The end date of June 2022 was used for censored observations. hPG_80_ levels below the threshold LoQ were imputed for 129 (out of 464) women in our study using a truncated Normal distribution via the R package *TruncNorm*. hPG_80_ values above the threshold LoQ were log transformed to generate a truncated Normal distribution that was used to generate log-transformed hPG_80_ values below the LoQ. These values were randomly assigned to the 129 women with missing values.

Clinically meaningful cut points of hPG_80_ levels that maximized each survival outcome were obtained using the R package *survminer*. Kaplan–Meier curves were generated for each survival outcome stratified by the low ( ≤) or high ( >) hPG_80_ group based on the respective hPG_80_ cut point. Adjustment for treatment (chemotherapy (Yes or No), radiotherapy (Yes or No), cancer stage (I, II, III) and age diagnosis (continuous scale) were included in Cox proportional hazards (PH) models for all survival outcomes that included log hPG_80_ levels measured on a continuous scale.

Menopausal status and age at diagnosis were assessed for their association with hPG_80_ including interactions. Sensitivity analyses were conducted by replacing age at diagnosis with menopausal status or a binary age at diagnosis; and hPG_80_ cut points replaced hPG_80_ measured on a continuous scale. Since this was a non-randomized study, cancer treatments (chemotherapy and radiation therapy) were forced in to provide a crude adjustment for their impact on survival outcomes. Cancer stage was the most significant tumour characteristic, had women with events at each level and never violated the proportional hazard (PH) assumption; it was also included in the final Cox PH models. Tumour grade and size did not meet at least one of these criteria so were not included. Proportional hazards assumptions were tested by assessing Schoenfeld residuals and Harrell’s C calculated to assess the final model prediction. Statistical significance was set at 0.05 for all statistical analyses. All analyses were conducted using Stata 17.

## Results

The 464 participants in this study were predominantly over 50 years of age (73.5%) and post-menopausal (75.4%). Most were diagnosed as a Luminal A subtype, as most had positive receptor status for estrogen (84.9%) and progesterone (78.2%), and negative receptor status for HER-2 (78.2%). Participants were approximately evenly split on participation in chemotherapy (49.3% did not participate, while 50.7% did participate), but were more likely than not to have undergone radiation therapy (65.3%), hormone therapy (75.9%), and surgery (100.0%), while being less likely to have undergone immunotherapy (8.41%). The most frequent stage at diagnosis was Stage I (52.2%) followed by Stage II (40.1%). Tumours were more likely to be graded as medium (41.2%) or high (40.3%), with an average tumour size of 20.8 mm (Standard Deviation (SD) = 14.3 mm). At the study end date, 50 women had experienced a recurrence of their breast cancer and 50 had died, including 29 who had a previous recurrence. Table 1 contains descriptive statistics relevant to our analytical population.

**Table 1:** Descriptive Statistics for Study Population (*N* = 464)

Variable	Values
	**Means (SD**^a^**)**
**hPG**_**80**_** (pmol/L)**	6.05 (27.88)
**Log- hPG**_**80**_** (pmol/L)**	0.98 (1.03)
**Diagnosis Age (years)**	57.04 (11.13)
**Tumour Size (mm)**	20.76 (14.32)
	**Frequency (percentage)**
**Diagnosis Age Group (years)**	
Under-50	123 (26.51)
50 +	341 (73.49)
**Menopausal Status**	
Pre-Menopausal	114 (24.57)
Post-Menopausal	350 (75.43)
**Vital Status**	
Alive	414 (89.22)
Deceased	50 (10.78)
Cause: Breast cancer	31 (62.00)
Cause: Other cancer	9 (18.00)
Cause: Other	10 (20.00)
**Estrogen Receptor**	
Positive	394 (84.91)
Negative	56 (12.07)
*Missing*	14 (3.02)
**Progesterone Receptor**	
Positive	363 (78.23)
Negative	86 (18.53)
*Missing*	15 (3.23)
**HER-2 Receptor**	
Positive	82 (17.67)
Negative	363 (78.23)
*Missing*	19 (4.09)
**Hormone Receptor Combinations**	
Triple Negative	30 (6.47)
HR^b^ Negative, HER-2 Positive	17 (3.79)
HR Positive, HER-2 Negative	330 (71.12)
HR Positive, HER-2 Positive	63 (13.58)
*Missing*	24 (5.17)
**Treatment Participation**	
Chemotherapy	
Yes	229 (49.35)
No	235 (50.65)
Radiotherapy	
Yes	303 (65.30)
No	161 (34.70)
Surgery	
Yes	464 (100.00)
Hormone Therapy	
Yes	352 (75.86)
No	112 (24.14)
Immunotherapy	
Yes	39 (8.41)
No	425 (91.59)
**Cancer Stage**	
I	242 (52.16)
II	186 (40.09)
III	36 (7.76)
**Tumour Grade**	
Low	71 (15.30)
Medium	191 (41.16)
High	187 (40.30)
*Missing*	15 (3.23)

^a^*SD* standard deviation

^b^*HR* hormone receptor; is negative if both Estrogen Receptor (ER) and Progesterone Receptor (PR) are negative; is positive if at least one of ER or PR is positive

**Fig. 1:**
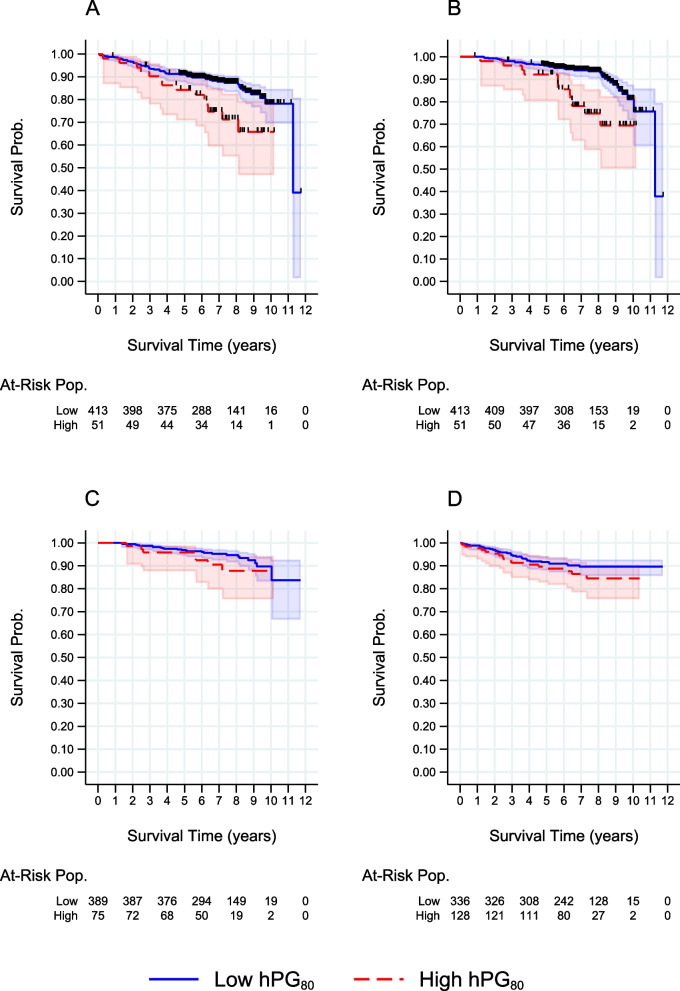
Kaplan–Meier Curves for Survival Outcomes by hPG_80_ Group Status based on Corresponding Cut Point Determination. Censored observations denoted by ‘ + ’, low hPG_80_ group is blue solid line, high hPG_80_ group is red dashed line. **a** Relapse-free survival, **b** Overall survival, **c** Breast – specific survival, and (**d**) Time – to – recurrence

Table 2 contains the summary of hPG_80_ cut point determinations found using the R package *survminer*. The same cut point value of 9.84 pmol/L was found for both recurrence-free survival (*p* = 0.009) and overall survival (*p* < 0.001), both of which were determined to be statistically significant using the Log-Rank test. A cut point of 6.77 pmol/L was found for breast cancer-specific survival (*p* = 0.183), and a cut point of 4.02 pmol/L was found for time to recurrence (*p* = 0.240), though neither were found to be statistically significant. Figure 1 contains the plotted Kaplan–Meier curves associated with these estimated hPG_80_ cut points for each survival outcome. The log rank tests were statistically significant for both time-to-recurrence (0.009) and overall survival (< 0.001) but not breast-cancer survival (0.183) or recurrence-free outcomes (0.240).

**Table 2:** Summary of hPG_80_ Cut point Determinations for each Survival Outcome (*N* = 464)

**Survival Outcome**	**hPG**_**80**_** Cut point (pmol/L)**	**Log- hPG**_**80**_** Cut point (pmol/L)**	**Log Rank Test Statistic (*p*-value)**	**N. Low Group**^**a**^	**N. High Group**^**b**^
Recurrence-free survival	9.84	2.286	2.526 (0.009)	413	51
Overall survival	9.84	2.286	3.216 (< 0.001)	413	51
Breast-specific survival	6.77	1.913	1.263 (0.183)	389	75
Time-to-recurrence	4.02	1.391	1.165 (0.240)	336	128

^a^ N. Low Group – Number of Participants below or equal to hPG_80_ cut point

^b^ N. High Group – Number of Participants above hPG_80_ cut point

After adjusting the Cox PH models for age at diagnosis, participation in chemotherapy, participation in radiation therapy, and cancer stage, hPG_80_ was estimated to be a significantly hazardous predictor for recurrence-free survival (HR: 1.399; 95% Confidence Interval (CI): 1.106 – 1.770; *p* = 0.005) and overall survival (hazard ratio (HR): 1.385; 95% CI: 1.046 – 1.834; *p* = 0.023). Additionally, hPG_80_ was also estimated to be a potentially hazard predictor for time to recurrence (HR: 1.330; 95% CI: 0.995 – 1.777; *p* = 0.054) but was not found to be a significant predictor for breast cancer-specific survival (HR: 1.015; 95% CI: 0.684 – 1.505; *p* = 0.942). Harrell’s C range from 0.64 to 070 indicating fair prediction based on these models (Table 3).

**Table 3:** Cox Proportional Hazards Regression Model Results by Survival Outcome

Variable	Recurrence-Free Survival HR (*p*-value) 95% CI	Overall Survival HR (*p*-value) 95% CI	Breast-Specific Survival HR (*p*-value) 95% CI	Time to Recurrence HR (*p*-value) 95% CI
**Log- hPG**_**80**_** (pmol/L)**	1.399 **(0.005)** **1.106 – 1.770**	1.385 **(0.023)** **1.046 – 1.834**	1.015 (0.942) 0.684 – 1.505	1.330 (**0.054)** **0.995 – 1.777**
**Participation in Chemotherapy**				
No	REFERENCE GROUP			
Yes	0.885 (0.717) 0.457 – 1.714	1.151(0.729) 0.521 – 2.541	1.630 (0.356) 0.578 – 4.600	1.158 (0.718) 0.523 – 2.565
**Participation in Radiotherapy**				
No	REFERENCE GROUP			
Yes	0.827 (0.477) 0.491 – 1.395	0.819 (0.540) 0.432 – 1.552	0.959 (0.926) 0.397 – 2.319	0.885 (0.707) 0.468 – 1.675
**Diagnosis Age (years)**	0.983 (0.171) 0.958 – 1.008	1.024 (0.148) 0.992 – 1.056	1.027 (0.191) 0.987 – 1.068	0.967 **(0.027)** **0.940 – 0.996**
**Cancer Stage**	Overall: ***p* = 0.005**	Overall: ***p* = 0.003**	Overall: ***p* = 0.001**	Overall: ***p* = 0.030**
Stage I	REFERENCE GROUP			
Stage II	1.599 (0.129) 0.872 – 2.931	1.452 (0.314) 0.703 – 3.003	1.000 (> 0.999) 0.378 – 2.645	1.352 (0.419) 0.651 – 2.804
Stage III	3.984 **(0.001)** **1.731 – 9.169**	5.004 **(0.001)** **1.933 – 12.952**	5.555 **(0.002)** **1.865 – 16.539**	3.373 **(0.011)** **1.314 – 8.655**
Harrell’s C-Index	0.639	0.652	0.695	0.675

^1^Reported data: estimated hazard ratio and p-value (top); 95% confidence interval (bottom)

Additionally, age at diagnosis was found to be significantly protective predictor for time to recurrence (HR: 0.967; 95% CI: 0.940 – 0.996; *p* = 0.027). Cancer stage was found to be a significant hazardous predictor for all 4 survival outcomes; patients diagnosed with stage III breast cancers were estimated to be at much greater risk than patients diagnosed with stage I cancers in recurrence-free survival (HR: 3.98; 95% CI: 1.73 – 9.17; *p* = 0.001), overall survival (HR: 5.004; 95% CI: 1.93 – 12.95; *p* = 0.001), breast cancer-specific survival (HR: 5.56; 95% CI: 1.87 – 16.54; *p* = 0.002), and time to recurrence (HR: 3.37; 95% CI: 1.31 – 8.66; *p* = 0.011).

The sensitivity analyses did not alter any of these findings for the final Cox PH model (results not shown). For instance, adding tumour grade to the base model (with or without stage) did not change the significance of hPG_80_ for each outcome. Similarly, substituting the continuous version of hPG_80_ with one based on outcome-specific cut points did not change the significance of hPG_80_ for each outcome. Adding breast cancer recurrence as time-varying factor in the overall survival model resulted in hPG_80_ having a slightly larger *p*-value of 0.066. The only exception was when menopausal status was substituted for age at diagnosis in the final model, it was statistically significant for recurrence-free and overall survival but not for time to recurrence.

## Discussion

Our study found significant associations between increasing hPG_80_ levels measured at diagnosis and the risk of recurrence or death in women with breast cancer. This risk was independent of chemotherapy or radiation therapy received, age at diagnosis or stage of disease. Older women at diagnosis had lower risks of recurrence and women diagnosed at Stage III were at substantially higher risk of all four outcomes. Additionally, cancer stage was found to be a significant predictor of increased hazard for all three outcomes – stage III in particular, while age at diagnosis was a significant predictor of decreased hazard for time-to-recurrence. Using the clinically-relevant cut point versions of hPG_80_ did not change the conclusions that were based on the continuous version.

A recent paper compared hPG_80_ blood levels from 11 different cancers to hPG_80_ levels from healthy controls [11]. The median hPG_80_ levels were significantly higher in the blood of cancer patients (4.88 pmol/L) than healthy blood donors (1.05 pmol/L) [11]. The results of our research also support the relationship of increased hPG_80_ levels and more cancer-related outcomes. For instance, the proportion of recurrences in the low hPG_80_ group was 16.3% versus 27.5% in the high hPG_80_ group (data not shown). The clinical cut point identified in our study for time to recurrence (4.02 pmol/L) was similar to those found in renal cell and hepatocellular carcinoma patients [19, 20]. The other cut points for the remaining three survival outcomes were higher. The study on renal cell carcinoma and hepatocellular carcinoma showed a significant association with median overall survival where the cut-off for hPG_80_ level was at 4.5 pmol/L for an approximate 12 month survival for both cancers; higher levels of hPG_80_ resulted in shorter survival [19, 20]. Additionally, the study on hepatocellular cancer suggest that the cohort exhibited a higher sensitivity to hPG_80_ than AFP, the common prognostic biomarker used for hepatocellular carcinoma, where combined measurement of hPG_80_ and AFP (clinical cut-point at 100 ng/mL) improved prognosis for patients with low AFP [20].

The hPG_80_ gene is the direct target of the WNT/β-catenin pathway – a pathway involved in the tumorigenesis of multiple organs [24]. The WNT/β-catenin pathway is associated with pluripotency, self-renewal of stem cells, and differentiation; however abnormal activation of the pathway promotes activation of cancer stem cell progression and hence, metastasis [25]. In colon carcinogenesis, the WNT/β-catenin pathway is further enhanced by excess hPG_80_ secretion and is considered an early marker of colon carcinogenesis [26]. Though the mechanism of the WNT/β-catenin pathway and hPG_80_ has primarily been studied in relation to colon cancer, similar mechanisms may also exist in other cancers, such as breast cancer.

Other novel biomarkers, still under evaluation in clinical studies, may also have the potential to determine prognostic outcomes of breast cancer. These potential biomarkers include circulating carcinoma proteins, circulating tumor cells, circulating cell-free tumor DNA, circulating microRNA (miRNA), extracellular vesicles, multi-analyte tests, and others [27]. The association between survival outcomes amongst some types of circulating carcinoma proteins are promising. For example, an increase in hepatocyte growth factors was shown in studies to be correlated with breast cancers as a risk factor and a high risk in metastatic progression [28]. However, interestingly, an increase in hepatocyte growth factors was paradoxically associated with long relapse-free survival [29]. In a systematic review and meta-analysis, circulating tumor DNA (ctDNA) was associated with a high risk of relapse; however, the review mentions there was heterogeneity of the studies, that all studies could not be included since relevant data were not available, and the studies used different techniques to quantify ctDNA [30]. Multiple types of circulating miRNA exist, and reviews suggest that high levels of circulating miRNA are associated with poor disease-free survival and prognosis of breast cancer [31]. Finally, a recent review suggests that extracellular vesicles are a potential biomarker for many cancers, including breast cancer [32]. Supporting this theory, one study on metastatic breast cancer patients found results suggesting extracellular vesicles are a potential predictor of progression free survival in metastatic breast cancer [33]. Compared to the above-mentioned new technologies, hPG_80_ is easily detectable in the plasma using ELISA technology and could be tested throughout the patient’s journey to potentially identify patients who may need a deeper biological assessment at an acceptable economic cost.

The major strength of this cohort is that it is prospective, minimizing the possibility of selection bias and differential misclassification. Additionally, hPG_80_ was measured at baseline before treatment began so any potential treatment effects on the hPG_80_ levels were avoided. The results of this study could be generalizable to women diagnosed with breast cancer, as the underlying biological mechanism should be the same for all individuals; however, the cohort was primarily Caucasian, preventing the evaluation in other ethnic groups. Biologically, African-American women have a higher frequency of grade 3 tumors than Caucasian women, and higher proportions of triple negative breast cancers [34]. Although the sample size for this study was nearly 500 women, very few women were diagnosed with a triple negative or HER-2 positive subtype limiting subgroup evaluations. Few women died from their breast cancer, likely contributing to low statistical power to detect an association with hPG_80_. Despite this limitation, the cohort is able to provide novel and greater insight into an important relationship between hPG_80_ and breast cancer.

## Conclusions

This is the first study observing the relationship between breast cancer outcomes and hPG_80_ levels. The prevalence of bone metastases in early stage breast cancers in Canada is 20–30%, providing a compelling need for better prognostic tools [7]. Future studies could focus on determining robust clinical cut points for all breast cancer subtypes, the provision of more customized treatments. Additionally, longitudinal measurements of hPG_80_ levels from diagnosis to events like recurrence or death could better elucidate this relationship.

## Data Availability

The datasets analyzed during the current study are not publicly.available due to limitations on the participant consents obtained for enrollment in the B2B study. Please contact Karen Kopciuk to enquire about data availability.

## Abbreviations

ACRB: Alberta Cancer Research Biorepository
AFP: Alpha-fetoprotein
B2B: Breast to Bone cohort
CA 15–3: Cancer antigen 15–3
CEA: Carcinoembryonic antigen
ctDNA: Circulating tumor DNA
EDTA: Ethylenediaminetetraacetic acid
ER: Estrogen receptor
Her 2: Human Epidermal Growth Factor Receptor 2
HR: Hazard ratio
LoD: Limit of detection
LoQ: Limit of quantitation
miRNA: MicroRNA
PH: Proportional hazards
PR: Progesterone receptor
SD: Standard Deviation
